# Effect of Ultrafiltration of Pitaya Extract (*Stenocereus thurberi*) on Its Phytochemical Content, Antioxidant Capacity, and UPLC-DAD-MS Profile

**DOI:** 10.3390/molecules25020281

**Published:** 2020-01-09

**Authors:** Daniela D. Castro-Enríquez, Beatriz Montaño-Leyva, Carmen L. Del Toro-Sánchez, Josué E. Juárez-Onofre, Elizabeth Carvajal-Millán, Guadalupe A. López-Ahumada, Carlos G. Barreras-Urbina, José A. Tapia-Hernández, Francisco Rodríguez-Félix

**Affiliations:** 1 Department of Food Research and Graduate Program, University of Sonora, Hermosillo 83000, Sonora C.P., Mexico; daniela.castro.e@hotmail.com (D.D.C.-E.); beatriz.montano@unison.mx (B.M.-L.); carmen.deltoro@unisonmx.onmicrosoft.com (C.L.D.T.-S.); amanda.lopez@unison.mx (G.A.L.-A.); carlosgbarrerasu@gmail.com (C.G.B.-U.); tapia_hernandez_agustin@hotmail.com (J.A.T.-H.); 2 Department of Physical, University of Sonora, Hermosillo 83000, Sonora C.P., Mexico; josue.juarez@unison.mx; 3 Research Center for Food and Development, A.C. Biopolymers-CTAOA, Hermosillo 83304, Sonora C.P., Mexico; ecarvajal@ciad.mx

**Keywords:** pitaya, *Stenocereus thurberi*, phytochemical, antioxidant capacity, ultrafiltration, UPLC-DAD-MS

## Abstract

*Stenocereus thurberi* is an endemic species in northwestern Mexico. It produces colorful fruits called pitayas that have an edible pulp. They have phytochemical compounds associated with biological activities. Ultrafiltration is a widely used method for the clarification of fruit juices and the recovery of phytochemicals. However, its effect has not been extensively studied in extracts. Therefore, the objective of this work is to study the effect of the ultrafiltration of pitaya extract (*Stenocereus thurberi*) on its phytochemical content, antioxidant capacity, and identification of phenolic compounds by UPLC-DAD-MS, providing greater knowledge about the pitaya. In this study, two extracts were analyzed, the unclarified extract (UE) and the clarified extract (CE). The antioxidant capacity was higher in the CE with 15.93 ± 0.42 mM TE/g, DPPH and 18.37 ± 0.016 mM TE/g, ABTS. The UPLC-MS analysis indicated the decrease in phenolic compounds in the CE and the presence of gallic acid and resorcinol, compounds that had not been identified in other species of *Stenocereus* spp. The correlation analysis indicated that all the phytochemicals present in the pitaya contribute significantly to the antioxidant capacity. The ultrafiltration process could be a viable option to improve the biological activity of the natural extracts.

## 1. Introduction

The columnar cactus *Stenocereus thurberi* is an endemic species in the northwest of Mexico and is little known compared to other species such as *Stenocereus queretaroensis*, *Stenocereus pruinosus*, and *Stenocereus stellatus* [1]. This genus produces prickly, spherical, or ovoid fruits commonly called pitayas, its flesh edible and of different colors which vary in shades of yellow, orange, red, and purple due to the presence of betalains [2].

Betalains are water-soluble nitrogenous pigments, and are synthesized from the amino acid tyrosine into two structural groups: betacyanins that represent a red-violet coloration, and betaxanthins with yellow-orange coloration [3]. These pigments present diverse biological activities, among which stand out are their anti-inflammatory, anticancer, and antioxidant properties [4]. The antioxidant activity of betalains is derived from the presence of betalamic acid, which is an aromatic amino compound capable of stabilizing free radicals. This stabilization is closely related to its ability to donate electrons [5]. Another important group of phytochemicals found in pitaya pulp are phenolic compounds, which, like betalains, have been associated with biological activities, which are attributed to the phenolic hydroxyl groups that are H-donor antioxidants eliminating reactive species. In this way phenolic compounds can inhibit the oxidation of lipids, proteins, DNA, and enzymes involved in the generation of radicals that are associated with chronic degenerative diseases, so we can say that these phytochemicals play an important role in nutrition and human health. [1]. Nowadays, the recovery of biologically active phytochemicals from natural sources has generated great interest due to its potential use as ingredients in food products, pharmaceuticals, and cosmetic formulations [6].

Ultrafiltration is a physical method of recovery and separation of compounds as of a feed solution under applied hydrostatic pressure, so the feed solution is divided into a permeate fraction containing all the compounds that permeate the membrane and a retention fraction that did not permeate the membrane. The process does not involve temperature, does not apply phase changes or chemical agents, and is characterized by high efficiency, low energy consumption, and simple equipment. For this reason, ultrafiltration is one of the most used methods in the food industry, mainly for the clarification of fruit juices and the collection of bioactive compounds [7,8]. In the literature, there are studies on the use of membranes for the extraction and separation of bioactive compounds from various natural extracts [9,10,11,12,13]. However, there are few studies on the effect of ultrafiltration on cacti fruit extracts, and research does not specify the molecules that are involved before and after the process [7,14,15,16]. Thus, a better understanding of the effect of ultrafiltration requires the use of chromatographic tools such as UPLC-DAD-MS to help explain why certain ultrafiltrated extracts are benefited or impaired in their biological activity. The objectives of this work are to study the effect of the ultrafiltration of pitaya extract (*Stenocereus thurberi*) on its phytochemical content, antioxidant capacity, and identification of phenolic compounds by UPLC-DAD-MS, as well as to provide greater knowledge on the processing of pitaya extract, given that it is a little known and valued source.

## 2. Results and Discussion

### 2.1. Physico-Chemical Characterization

**Figure 1:**
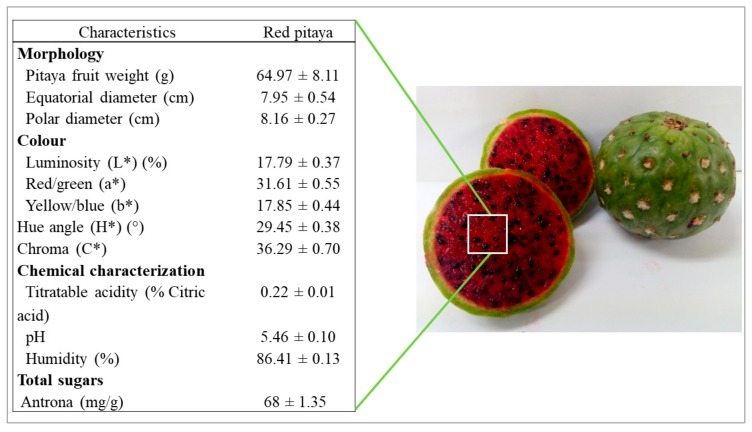
Physical-chemical composition of pitaya pulp (*Stenocereus thurberi*).

Figure 1 shows the physical and chemical characteristics of the red pitaya of *S. thurberi*. The morphological attributes of the fruit show an almost spherical shape with polar and longitudinal diameters larger than those reported for *S. pruinosus*, *S. stellatus,* and *S. griseus*; however, the fruit of *S. thurberi* presented less weight compared to the fruits of the species mentioned above, ranging from 171.15 g to 91.26 g. The differences in weight between the fruits of these species are due to their high perspiration rate and the number of areolas, since the thorns present in the areolas help to reduce water loss [2]. Transpiration is one of the causes of post-harvest losses of this fruit and is classified as a phenomenon of water transport from the inside of the fruit to the outside, through plant structures such as the epidermis [17]. Regarding its chemical attributes, the amount of total sugars was similar to that reported for *S. pruinosus* (57.1–66.7 mg/g), *S. stellatus* (60.7–68.6 mg/g), and *S. griseus* (76.0–103.0 mg/g) [2,18], suggesting that such content could be a characteristic feature of the *Stenocereus genus* [18]. The titratable acid value of the red pitaya *S. thurberi* was similar to that reported for *S. pruinosus* (0.17%) and *S. griseus* (0.18%), while in *S. stellatus* are reported values of 0.60%, consistently the pH value was similar to that reported for *S. pruinosus* (5.70), while for *S. stellatus*, values of 4.19 are reported [2], suggesting that pitaya fruit *S. thurberi* is sweeter that fruit *S. stellatus*. In terms of the color attributes of the red pulp of *S. thurberi*, values of 29.45° were obtained for the hue angle, 36.29 for chroma, and 17.19% for luminosity. Comparing these results with what was reported by García et al. [19] where they evaluated the color for the red pulp of *S. pruinosus* (L* = 19.4%, H* = 19.8° and C* = 24.7), *S. thurberi* it has a red-orange hue, while *S. pruinosus* has a magenta hue. The color of the pitaya fruit is derived from the presence of betalains [20], whereby the color is an indicator of the proportion between the two different groups (betacyanins and betaxanthins); therefore, it can be assumed that pulp of *S. pruinosus* has a higher betacyanins content than betaxanthins, while in the pulp of *S. thurberi*, the proportion could be very similar between both groups.

### 2.2. Phytochemical Content

Table 1 shows the phytochemical content of the UE and CE of pitaya. The content of total phenols for the UE was similar to that reported for the fruits of other cactaceae such as garambullo (*Myrtillocactus geometrizans*) with values of 7.4 to 10.02 mg GAE/g [21] and Cuaresmeño xoconostle (*Opuntia matudae*) with 8.5 mg GAE/g [22]. However, for other genera and species of cactus such as *O. stricta*, *O. undulara,* and *O. ficus indica*, lower values of 1.64 to 2.18 mg GAE/g have been reported [23,24]. García et al. [2], reported the total phenolic content of the *S. pruinosus* and *S. stellatus* of red, orange, and white pitaya pulp with values of 0.35–0.70 mg GAE/g mentioning that the red variety was the one that presented the value higher. While in the pitaya pulp *S. griseus* was 1.66 mg GAE/g [19]. The content of phenols is variable among the fruits of cactaceaes, even of the same genus, showing greater content in the red pulp of pitaya *S. thurberi*, studied by us. However, these data could be overestimated, because the colorimetric method used in its determination is not specific for phenols, and other compounds such as sugars and vitamin C can reduce the Folin-Ciocalteu reagent [25], which can be confirmed with the total sugar values mentioned above and titratable acidity. The total phenolic content showed significant differences (*p* < 0.05) between the analyzed extracts, with 13.89 ± 0.75 and 20.70 ± 0.81 mg GAE/g in UE and CE, respectively, a result consistent with those reported by Vergara et al. [26] where they physicochemically evaluated the ultrafiltered and unfiltered extract of the prickly pear (*O. ficus indica*), finding significant differences in the quantification of phenols in both extracts. Ultrafiltration is a method of separation of molecules, and in its use in the fruit extracts of cacti, it has been observed that molecules such as polysaccharides, proteins, and sugars are diminished; incidentally this result may be due to the greater availability of reactive groups that can reduce reagent Folin-Ciocalteu, contributing to a greater quantification of phenols [26,27].

The content of betacyanins and betaxanthins was higher compared to prickly pear (*O.* *ficus indica*) where for betacyanins it ranges from 0.07 to 0.254 mg/g and betaxanthins 0.12 to 0.88 mg/g [26,28]. In pitayas of species such as *S. pruinosus* and *S. stellatus*, betalain values of 0.535 mg/g and 0.707 mg/g, respectively, have been reported [2]. Pérez et al. [29] analyzed the betalains content in *Stenocereus stellatus* pitayas and compared it with 32 samples of other cactus fruits in order to classify cactus fruits as poor, good, and excellent sources of betalains They calculated three confidence intervals for the total betalain content, considered a low (lower 1208 μg/g), medium (2935–3288 μg/g) and high (4488–9248 μg/g). Considering this classification, the betalains content in the pitaya pulp *Stenocereus thurberi* is defined as a good source of betalains. The beet *(Beta vulgaris*) has a betalains content of 39.76 mg/g, well above the values obtained with respect to other sources [30]. However, pitaya fruits have higher concentrations of betalains compared to other cactaceae. On the other hand, the betalains content in both extracts did not show significant differences. These results are consistent with those reported by Castro et al. [27] and Vergara et al. [26], who evaluated the physicochemical characteristics of the ultrafiltered extract of the prickly pear (*O. ficus indica*) where they found no significant differences in betalains content between both extracts. The slight decrease in betalains in the UE is due to the turbidity caused mainly by the presence of polysaccharides causing an increase in color intensity [27].

### 2.3. Antiradical Capacity ABTS ^+^ and DPPH^·^

**Figure 2:**
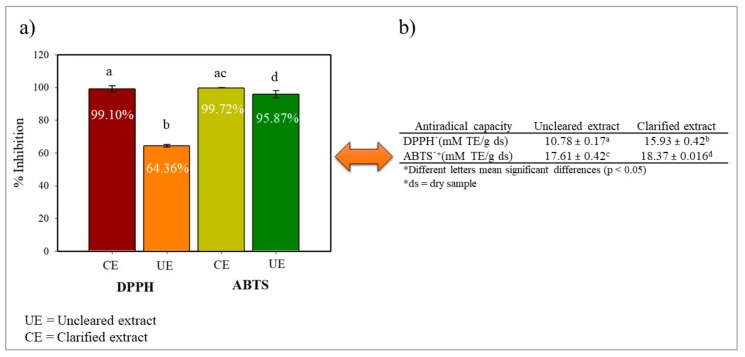
Antiradical capacity ABTS^·^^+^ and DPPH^·^ (**a**) presented in percent inhibition and (**b**) in Trolox equivalents of the UE and CE of pitaya (*Stenocereus thurberi*).

Figure 2a,b shows the antioxidant capacity of UE and CE by the DPPH and ABTS methods expressed in % inhibition and mM TE/g of dry samples. We can observe that both extracts showed great antioxidant capacity, comparing these results with other cactaceous fruits. García et al. [2] evaluated by ABTS the antioxidant capacity of the pitaya pulp (*S. pruinosus* and *S. stellatus*) of different colors: red (Spr, Ssr), orange (Spo), and white (Ssw), respectively. They mention that the red pitayas for both species had the highest antioxidant capacity with values for Spr 4.91 ± 0.20 mM TE/Kg and Ssr 6.68 ± 072 mM TE/Kg of dry samples. Another of the fruits of cacti that have been evaluated by ABTS is the prickly pear (*Opuntia ficus-indica*), which shows an antioxidant capacity in a range of 0.0337–0.0856 mM TE/g of fresh samples [31,32]. In the red pitaya (*Hylocereus polyrhizus*) the antioxidant capacity with the DPPH radical was also determined, the value obtained being 0.001664 mM TE/g [33]. These results may be due to the presence of phenolic compounds and betalains capable of reducing DPPH and ABTS radicals. The identification of phytochemicals of the genus *Stenocereus spp* from two species *S. pruinosus* and *S. stellatus* has been reported in the literature. García et al. [1] reported that the profiles and concentrations of betalains and phenolic compounds in the fruits of the two *Stenocereus species* studied, varied according to the color of the pulp and among the species. In addition, in the red varieties, betalains were present at much higher levels than the phenolic compounds. Betalains are molecules with a high antioxidant capacity comparable to epicatechin gallate, capable of donating electrons and protons due to the presence of a 5-O-glucosyl catechol and 1,7-diazaheptamethinium [34]. However, not all betalains have the same antioxidant capacity, and this will depend on the source and position of the hydroxyl/imino groups and the glycosylation of aglycone in the betalains structure [35].

On the other hand, the antioxidant capacity was significantly different between the extracts and in both radicals. These results are consistent with those reported by Castro et al. [7] where antioxidant capacity values are favored by ultrafiltering the extract, they obtained values of the unfiltered extract of 11.48 µM TE/g and ultrafiltrate 15.09 µM TE/g in the prickly pear extract (*Opuntia ficus-indica*). In the literature, it has been reported that the purified extracts the potential of its functional properties was increased. Tenore et al. [36] evaluated the complete and purified extracts of pitaya (*Hylocereus polyrhizus*) in microorganisms and they mentioned according to the values of the minimum inhibitory concentration (MIC) that the purified extracts of both pulp and shell exerted a broad antimicrobial spectrum by inhibiting the growth of all bacteria, while whole extracts revealed very low activity or were totally inactive. Therefore, these results could be due to the permeate and concentration of highly antioxidant compounds present in the CE and in the UE may contain compounds that could act as prooxidants avoiding the reduction of radicals since it has been seen that with the ultrafiltration method there may be a retention of compounds in the membrane of up to 50% [14,37,38].

Table 2 shows the values of the concentration at which 50% of the reduction of ABTS and DPPH radicals is had. We can observe that the CE was the one that presented significantly lower values of IC 50 with respect to the UE. Luo et al. [39] reported IC50 values in the DPPH radical of 0.83 mg/mL for the *Hylocereus polyrhizus* pitahaya shell. However, the values obtained in the ABTS radical for CE are similar to those obtained for ascorbic acid with an IC50 of 1.81 mg/mL, which is a potent antioxidant [40]. The significant difference between the ABTS and DPPH radicals in both extracts may be due to the fact that the mechanism of inhibition used by both extracts is by hydrogen atom transfer (HAT) because of the inhibition of the ABTS radical predominates. However, this may be due to several factors such as solubility, link dissociation energy, ionization potential, antioxidant structure, and solvent [41].

### 2.4. Identification of Phenolic Compounds by UPLC-DAD-MS

**Figure 3:**
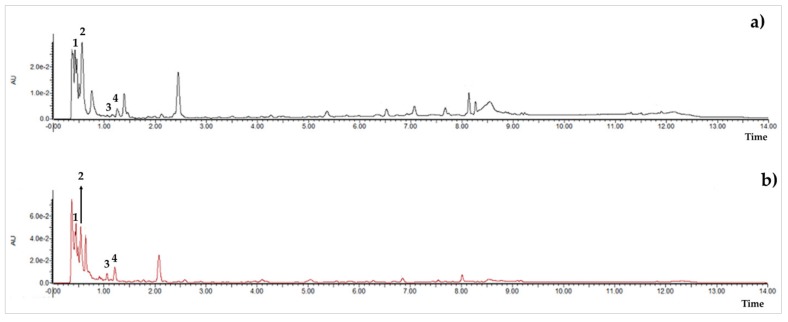
UPLC chromatograms of the phenolic compounds present in the uncleared extract (**a**) and clarified (**b**) at 280 nm, [y axis = intensity (absorbance unit, AU); x axis = retention time (min)]. Peaks: 1, ferulic acid; 2, gallic acid; 3, resorcinol and 4, catechin.

**Figure 4:**
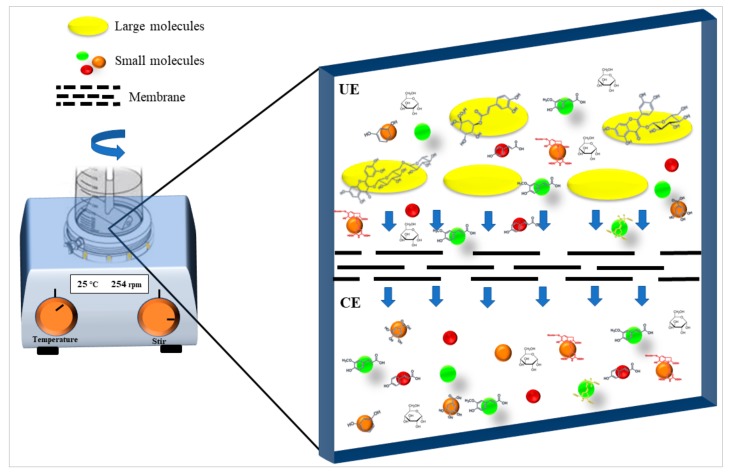
Scheme of the ultrafiltration process representing the main molecules present in the pitaya extract (*Stenocereus thurberi*) before and after the process.

Table 3 shows the results of the MS analysis with the possible compounds that are present in the samples. We can observe that in the UE there was a greater presence of phenolic compounds such as ferulic acid, caffeic acid, p-coumaric acid and quercetin with retention times of 0.43, 2.45, 4.19, and 9.23 min, respectively (Figure 3a). The presence of these phenolic compounds has also been reported by Garcia et al. [1] in the pitaya pulp of *Stenocereus stellatus* and *Stenocereus pruinosus*. However, in the CE there is a decrease in phenolic compounds (Figure 3b), mainly those that have the longest retention time such as caffeic acid, rutin, quercetin, and isorhamnetin to mention a few. This result can be related to the recovery factor of the ultrafiltration process where 50% ± 0.066 was obtained. Some researchers have studied the effect of ultrafiltration as reported by Pap et al. [40] who studied the effect of ultrafiltration on the content of anthocyanins and flavonoids in blackcurrant juice, the authors mention that the ultrafiltration process had a significant effect on the 50% decrease of these compounds compared to the initial juice. Also, Cassano et al. [42] observed that the decrease of phenolic compounds in the tangerine game, as well as the decrease of flavonoids in prickly pear juice [14]. This decrease is mainly due to the pore size of the ultrafiltration membrane to one that is not the only reason, but also to the phenomenon of membrane fouling, such as pore blockage and the formation of compound layers, which promote retention of compounds in the membrane [38]. In Figure 4, the ultrafiltration process is schematized, where it can be seen that the EC has a decrease in molecules. However, despite the decrease in phenolic compounds due to ultrafiltration in the CE, this extract showed a greater antioxidant capacity in both radicals. This indicates that in purified extracts functional properties can be favored, due to concentration, and that many compounds can act antagonistically or synergistically [36,43].

In both studied extracts of pitaya pulp (*Stenocereus thurberi*), the presence of gallic acid and resorcinol was identified at 0.56 min and 1.15 min, respectively (Table 3 and Figure 3a,b). It is our knowledge that such compounds have not been reported in the genus of *Stenocereus spp*. It is clear that there are few studies on the species of this genus and its fruits. Therefore, this finding could provide added value to the pitaya *Stenocereus thurberi*, since gallic acid has properties such as antimutagenic, antifungal, and is a potent antioxidant even more effective than ascorbic acid [44,45]. Similarly, resorcinol is a phenolic compound widely used in cosmetics, medications, and as an antioxidant [46].

### 2.5. Correlation Analysis between Phytochemicals and Antioxidant Capacity

In Table 4 we can observe the correlation coefficient values between the antioxidant capacity and the phytochemical content of the pitaya (*Stenocereus thurberi*). The correlation coefficients between antioxidant capacity and total phenolic content were positive and highly significant (*p* ≤ 0.01) for the DPPH radical and significant (*p* ˂ 0.05) for the ABTS radical in both samples. This observation is consistent with the work of Dehbi et al. [47] regarding the correlation of the total phenolic content with the antioxidant capacity in juices of nine prickly pear cultivars (*Opuntia ficus-indica*).

The antioxidant capacity of phenolic compounds is attributed to their redox properties, which allow them to act as reducing agents, hydrogen donors, oxygen inhibitors, and metal chelation [48]. The correlation coefficients between antioxidant capacity and betacyanins and betaxanthins content were greater than 0.90 (*r* ˃ 0.90) in all cases. The betacyanins and betaxanthins content of the pitaya was positively correlated with the antioxidant capacity and highly significant (*p* ≤ 0.01) for both radicals and samples, respectively. It has been reported that the antioxidant activity of betalains is related to its chemical structure since the antioxidant capacity increases with the number of hydroxyl groups and imino groups and decreases with higher glycosylation in the molecule [49]. However, according to Gandía et al. [50], the antioxidant activity of betalains, in particular betaxanthins, is not related to the presence of hydroxyl groups or aromaticity of the structure. According to the same authors, there is an “intrinsic activity” of betaxanthins that could be associated with the resonance system that is supported by the two nitrogen atoms and be general for all betalains. However, the presence of hydroxyl groups in betaxanthins increases their antioxidant capacity [50]. Therefore, these data suggest that phenolic compounds such as betacyanins and betaxanthins present in pitaya (*Stenocereus thurberi*) contribute significantly to antioxidant capacity.

## 3. Materials and Methods 

### 3.1. Raw Material

Pitaya fruits (*S. thurberi*) were randomly collected in the months of June–July 2016 in the township of Carbó, located to the west of the state of Sonora, Mexico between the geographical coordinates 29° 41′ north latitude, and 110° 57′ west longitude.

### 3.2. Reagents

The reagents used were ethanol (CTR, Monterrey, MX), methanol (Meyer, Monterrey, MX), 2, 2-Diphenyl-1-picrylhydrazyl (DPPH) (Sigma-aldrich, St. Louis, MO, USA), 2,2-azino-bis(3-ethylbenzothiazoline-6-sulfonic acid) (ABTS) (Sigma-aldrich, Oakville, CA), Folin-Ciocalteu’s phenol (Sigma-aldrich, St. Louis, MO, USA), trolox 6-hydroxy-2,5,7,8-tetramethylchromane-2-carboxylic acid (Sigma-aldrich, Steinheim, DE), gallic acid (Sigma-aldrich, Hong Kong, CN).

### 3.3. Physico-Chemical Characterization

Equatorial and polar diameters were evaluated with a Vernier gauge. Color was evaluated with a Hunter Lab colorimeter (Mini Scan XE Plus, Reston, Virginia, USA) and was expressed in hue angle (H*), chroma (C*), luminosity (L*), a* (verde/rojo) y b* (azul/amarillo). The pH was measured with potentiometer (Hanna, HI 2550), it was performed by weighing 1 g of pitaya pulp without seeds and 10 mL of milli-Q water was added (AOAC 981.12) [51]. Acidity was assessed by titration with NaOH 0.1N, performed by the weighing of 1 g of seedless pulp; 30 mL of mili-Q water was added, the solution was vigorously stirred, and subsequently the sample was titrated (AOAC 935.57) [51]. Total sugar content was evaluated with the method of antrona following the modified methodology of Laurentin and Edwards [52].

### 3.4. Preparation of Uncleared Extract and Clarified Extract from S. thurberi Fruits

Pitya fruits were washed and frozen at −20 °C for later use. The amount of 2 g of seedless pulp were macerated in 34 mL of distilled water. The mixture was placed in a water bath and subsequently sonicated (Branson, M3800H, Mexico city, MX) for 27 min and stirred for 20 min on a horizontal shaker (VWR, mini blot mixer, US) in the dark. Subsequently, the samples were centrifuged (Eppendorf, 5804 R, Hamburgo, DE) at 5000 rpm for 10 min. The supernatant obtained was lyophilized and stored in amber colored vials for later analysis. This sample corresponds to the uncleared extract (UE).

**Figure 5:**
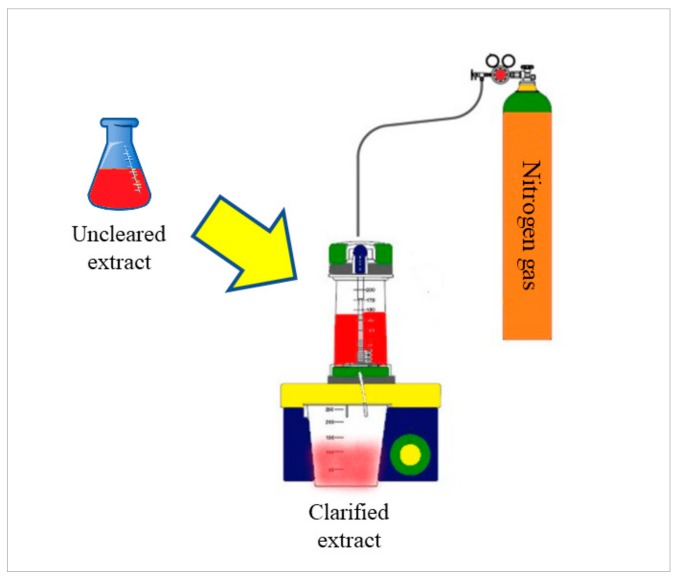
Ultrafiltration process to obtain the clarified extract.

For obtaining the clarified extract (CE), 15 mL of UE was used and passed through the ultrafiltration process. They were placed in the Amicon stirred cell with a capacity of 50 mL (Millipore, model 8050, Darmstadt, DE) using membranes (Millipore, Temecula, USA) with a 1 kDa molecular weight cutoff at 25 °C and a pressure of 50 psi of nitrogen gas (Figure 5). Subsequently, the CE was lyophilized and stored in amber colored vials for later analysis. For each sample, the analysis was performed in triplicate, and in each sample the membranes were washed with 10% (v/v) ethanol and mili-Q water. The following equation was used to obtain the recovery factor of the ultrafiltration process:

(1) $$\mathrm{RF}=\left(\frac{\mathrm{CE}}{\mathrm{UE}}\right)\;\times \;100$$

where CE is clarified extract and UE is uncleared extract.

### 3.5. Photometric Quantification of Betalains

The quantification of total betalains, betaxanthins, and betacyanins was carried out by measuring the absorbance at 538 nm for betacyanins and 483 nm for betaxanthins, using a UV-Vis spectrophotometer (Varian, Cary 50, Melbourne, AU). The amount of betalains was calculated by the following equation.

(2) $$\left[B\;\left(\frac{mg}{g}\right)\right]=\frac{A\times DF\times MW\times V}{\varepsilon \times W\times L}$$

where [B] means the betacyanins or betaxanthins concentration, A is absorbance of the sample, DF is dilution factor, MW is molecular weight (betanin 550 g/mol) (indicaxanthin 308 g/mol), V is volume, ε is molar extinction coefficient, for betanin it is 60,000 mol/L × cm and for indicaxanthin 48, 000 mol/L × cm, W is the weight of the sample (g) and L is the length of the cuvette (1 cm). The total betalains was obtained with the sum of betacyanins and betaxanthins.

### 3.6. Total Phenols

Determination of total phenols was carried out with the Folin–Ciocalteu (FC) method, where 10 μL of sample was mixed with 25 μL of FC 1 N and allowed to stand 5 min. After, 25 μL of 20% Na2CO3 and 140 μL of distilled water were added. It was allowed to stand for 30 min and the absorbance at 760 nm was determined. The results were expressed as milligrams of gallic acid equivalents for gram of dry sample (mg GAE/g).

### 3.7. Antiradical Capacity Using the ABTS ^+^ Assay

The stabilization of the ABTS ^+^ radical was determined using the method of Re et al. [53]. It was 270 μL of the radical solution with 20 μL of sample was added and allowed to stand 30 min in the dark. Absorbance was measured at 734 on a microplate reader (Thermo scientific, Multiskan go, Vantaa, Finland).

### 3.8. Antiradical Capacity Using the DPPH^·^ Assay

The stabilization of the DPPH radical was performed based on the method proposed by Molyneux [54]. It was 200 μL of radical with 20 μL of sample was mixed and allowed to stand for 30 min in darkness. The absorbance was measured at 515 nm on a microplate reader (Thermo scientific, Multiskan go, Vantaa, Finland). The results were expressed for both radicals in mM TE/g and % inhibition according to the following equation:

(3) $$\%inhibition=\frac{A_{o}-\left(A_{sample}-A_{blank\;sample}\right)}{A_{o}}\;\times \;100$$

where: $A_{o}$ is absorbance of reagent + H_2_O, $A_{sample}$ is absorbance of reagent + sample, $A_{blank\;sample}$ is absorbance of H_2_O + sample. From the % inhibition, the IC_50_ was obtained.

### 3.9. Identification of Phenolic Compounds by UPLC-DAD-MS

The identification of the phenolic compounds of the extracts of the pitaya pulp was made from a Waters UPLC analytical system (Waters, ACQUITY. Singapore) equipped with a diode array detector coupled to a mass spectrometer. For the analysis of the samples, a C18 column of 1.7 μm (2.1 × 50 mm) (ACQUITY, UPLC BEH) was used. The gradients used were 0.1% acetic acid in deionized water (A), methanol (B), and acetonitrile (C). An isocratic gradient of 90% (A), 5% (B) and 5% (C) was applied for 5 min, and then it was 78% (A), 11% (B), and 11% (C) for 5 min, 1 min of 36% (A), 31% (B) and 31% (C), and at the end a gradient of 90% (A), 5% (B) and 5% (C) was used for 2 min. The flow rate was 0.3 mL/min, the column and sample temperatures were maintained at 35 °C and 20 °C. The injection volume of the sample was 5 μL, and the absorbance was monitored at 280 nm.

Electrospray ionization (ESI) was operated in positive and negative ion modes. The mass spectra were obtained in a range of 100–750 *m/z*, the capillary voltage was 3.00 kV and a cone voltage of 30 V. The ESI-MS parameters were a desolvation temperature of 400 °C and a gas flow of 650 L/h. The identification of phenolic compounds was based on standards, *m/z* values, and scientific articles.

### 3.10. Statistical Analysis

The results of three independent tests are reported as the mean value ± standard deviation. Means were compared by analysis of variance and Tukey comparison test, with a significance level (*p* < 0.05). For the study of the degree of the linear relationship between the variables, the Pearson correlation coefficient was used. All the data were processed and analyzed in the InfoStat software by window version 2008 (InfoStat, Cordoba, Argentina).

## 4. Conclusions

The effect of ultrafiltration on phytochemical content, antioxidant capacity, and the profile of phenolic compounds in pitaya extract was studied. The content of phenolic compounds present in the pitaya extract *Stenocereus thurberi* was first identified by the UPLC-DAD-MS method. It was possible to identify gallic acid and resorcinol, compounds that have not been reported in other extracts of cacti. The use of the ultrafiltration method helped the separation, selection, and concentration of molecules, showing effects on antioxidant capacity, total phenolic content, and the profile of phenolic compounds. The ultrafiltration is a technology widely used at the industrial level that could help to potentiate the biological activity of natural extracts such as the pitaya (*Stenocereus thurberi*).

## Figures and Tables

**Table 1:** Phytochemical content in the clarified and uncleared extract of pitaya (*Stenocereus thurberi).*

Phytochemical		UE	CE
Phenols (mg GAE/g)		13.89 ± 0.75 ^a^	20.70 ± 0.81 ^b^
Betalains (mg/g)	Betacyanins	0.94 ± 0.15 ^a^	1.17 ± 0.27 ^a^
	Betaxanthins	1.16 ± 0.30 ^a^	1.37 ± 0.41 ^a^

* Different letters mean significant differences (*p* < 0.05).

**Table 2:** Values necessary to inhibit 50% of the DPPH^·^ and ABTS ^·^^+^ radical of the uncleared extract and clarified extract.

**IC_50_ (mg/mL)**		**Uncleared Extract**	**Clarified Extract**
	DPPH^·^	8.9 ^a^	5.6 ^c^
	ABTS^·^^+^	3.0 ^b^	1.8 ^d^

* Different letters mean significant differences (*p* < 0.05).

**Table 3:** Identification of possible phytochemicals present in the pitaya pulp (*Stenocereus thurberi*) of the UE and CE.

Peak		Chemical Structure	MF	MM	*m/z* (M + H)^+^	*m/z* (M-H)^−^	UE (Rt)	CE (Rt)
	Phenolic compounds							
1	Ferulic acid	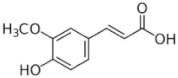	C_10_H_10_O_4_	381	382	380	0.43	0.45
2	Gallic acid	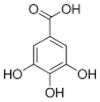	C_7_H_6_O_5_	170	171	169	0.56	0.56
3	Resorcinol	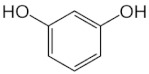	C_6_H_6_O_2_	110	111	N.D.	1.15	1.15
4	Catechin	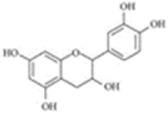	C_15_H_14_O_6_	290	291	289	1.21	1.25
5	Caffeic acid	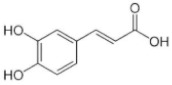	C_9_H_8_O_4_	180	181	N.D.	2.45	N.D.
6	p-coumaric acid	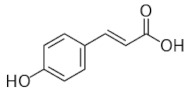	C_9_H_8_O_3_	164	165	163	4.19	4.19
7	Rutin	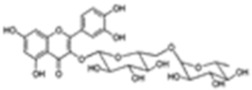	C_27_H_30_O_16_	610	611	609	6.52	N.D.
8	Isorhamnetin	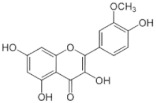	C_16_H_12_O_7_	316	317	N.D.	8.14	N.D.
9	Quercetin	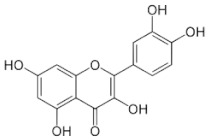	C_15_H_10_O_7_	302	303	N.D.	9.23	N.D.
10	Caffeoylquinic acid	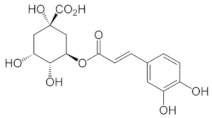	C_16_H_18_O_9_	354	N.D.	353	11.31	N.D.
11	Glycosylated quercetin	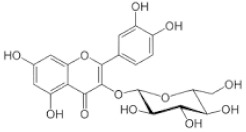	C_21_H_20_O_12_	464	465	N.D.	11.90	N.D.

N.D. = Not detected; RT = Retention time; MF = Molecular formula; MM = Molecular mass; *m/z* = mass/charge.

**Table 4:** Values of correlation coefficients between antioxidant capacity (ABTS, DPPH) and the phytochemical content of the Uncleared extract and Clarified extract of pitaya (*Stenocereus thurberi*).

Phytochemical		Coeficientes de Correlación (r)			
		UE		CE	
		* ABTS	* DPPH	* ABTS	* DPPH
Phenolic compounds		0.983	0.997	0.922	0.988
Betalains	Betacyanins	0.982	0.992	0.902	0.997
	Betaxanthins	0.991	0.994	0.941	0.991

* All data were significant (*p* < 0.05).
